# The Benefit of Being Proactive: The Effect of Proactive Personality on Professional Identity Among Chinese College Students

**DOI:** 10.3390/bs16040524

**Published:** 2026-04-01

**Authors:** Weilin Su, Xiao Gui, Huiyuan Chen

**Affiliations:** 1 School of Literature, Capital Normal University, Beijing 100089, China; suweilin@cnu.edu.cn; 2 School of Economics and Management, University of Science and Technology Beijing, Beijing 100083, China; m202511032@xs.ustb.edu.cn

**Keywords:** proactive personality, future self-continuity, career calling, professional identity, social cognitive career theory, college students

## Abstract

Based on social cognitive career theory, this study constructs and empirically tests a double-mediation model to elucidate the effect of proactive personality on professional identity among Chinese college students. Additionally, it delves into the mediating mechanisms underlying future self-continuity and career calling in this relationship. This study employs a three-stage data collection strategy, collecting data from 382 college students at a normal university in China. Statistical analysis results show that the proactive personality of college students has a significant positive impact on their future self-continuity, career calling, and professional identity. Meanwhile, future self-continuity and career calling of college students both play a mediating role in the influence of proactive personality on professional identity. In conclusion, the results of this study provide the academic community with a new perspective to explain the relationship between proactive personality and professional identity and can also provide specific guidance for improving college students’ professional identity in practice.

## 1. Introduction

Cultivating high-caliber talent through interdisciplinary and emerging disciplines has become a core focus for universities to maintain their global competitiveness (G. Liu et al., 2025). However, despite increased investment in higher education, the mismatch between university majors and graduates’ professional outcomes remains a significant challenge, leading to severe employment difficulties (Awsumb et al., 2022). A key factor contributing to this mismatch is the underdeveloped professional identity of college students (Wang et al., 2025). Numerous previous studies have also confirmed that insufficient professional identity can lead to cognitive dissonance, reduced engagement in learning, and ultimately produce a workforce that is technically skilled but psychologically disconnected from its profession (X. Liu et al., 2023; Tomlinson & Jackson, 2021). Enhancing professional identity among college students is crucial for their personal career success. Moreover, it plays a vital role in improving the overall effectiveness of the higher education system (Sweitzer, 2009).

Personality traits fundamentally shape how students interpret their academic majors and envision future careers (De Jong et al., 2019; Park et al., 2020). As a stable disposition, proactive personality enables individuals to act with environmental independence, transcending situational constraints (De Clercq & Pereira, 2023; Fuller & Marler, 2009). Based on social cognitive career theory (Lent et al., 2002), this study proposes that proactive personality influences professional identity through two parallel pathways: future self-continuity and career calling. Future self-continuity refers to the psychological connection between an individual’s future self and present self (Hershfield, 2011; Han et al., 2025), while career calling represents the strong and meaningful passion for a particular professional field (Dobrow & Tosti-Khabras, 2011; Yu et al., 2022).

**Figure 1:**
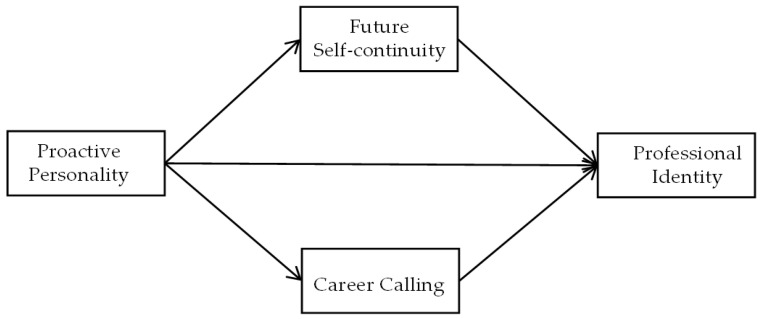
Theoretical research framework.

Building upon the theoretical foundations of social cognitive career theory and recognizing a significant research gap concerning the relationship between proactive personality and professional identity, this study aims to delve into the effects of college students’ proactive personality on their professional identity. Specifically, this study endeavors to address the following pivotal questions: (a) How does proactive personality influence professional identity among Chinese college students? (b) In what ways do future self-continuity and career calling serve as mediating mechanisms in the relationship between proactive personality and professional identity? (c) When compared to existing models grounded in social cognitive career theory, what distinctive contributions does our proposed dual-mediation model offer in elucidating the impact of proactive personality on professional identity? To enhance clarity and facilitate a deeper understanding of our proposed model, Figure 1 presents a theoretical framework diagram that visually articulates the interconnections among the core variables under investigation.

## 2. Theoretical Background and Hypotheses

### 2.1. Proactive Personality and Professional Identity

Rooted in the proactive behavior paradigm (Bateman & Crant, 1993), proactive personality is defined as a dispositional tendency to identify opportunities and take initiative in altering the environment (Major et al., 2006). It represents a person-environment fit where individuals actively shape rather than passively adapt to their context (Yu et al., 2023). Specifically, individuals with a strong proactive personality exhibit a lower sense of environmental constraints and a higher degree of behavioral autonomy (Seibert et al., 2001). They are usually able to anticipate potential changes, mobilize resources to take action, overcome obstacles, persevere, and ultimately achieve the expected results (F. Yang & Chau, 2016). In the context of higher education, this proactive quality enables students to go beyond passive learning and actively construct meaning for their own professional field (Lin et al., 2014).

To be specific, college students with a highly proactive personality typically do not passively accept course content but actively scan for career-related information in their environment, connecting current academic tasks with future career goals (Hu et al., 2021). This anticipatory role construction reduces role ambiguity and establishes a clear cognitive map of their future career (Hirschi et al., 2013), thus laying a solid foundation for their professional identity. At the same time, in the process of daily learning, they are able to actively explore their own strengths and enhance their professional self-efficacy through successful experiences (He et al., 2021), thus showing more proactive career exploration behaviors and a higher level of professional identity (Hsieh & Huang, 2014). Therefore, the first hypothesis of this study is proposed:

**Hypothesis** **1.** *Proactive personality of college students is positively related to their professional identity.*

### 2.2. The Mediating Role of Future Self-Continuity

Future self-continuity refers to the psychological connection between an individual’s future self and present self, representing the degree to which an individual views their future self as the same person as their present self (Hershfield, 2011). It is manifested in the degree of recognition of the future self and the ability to clearly define the present and future selves (Sokol & Serper, 2020). Many previous studies have shown that individuals with a high degree of future self-continuity tend to make choices that are beneficial to themselves in the long run and are more inclined to choose delayed gratification in decision-making (Wei et al., 2025). Meanwhile, they are also more likely to invest in their future selves and pay more attention to both their present and future selves (Hong et al., 2024).

For college students with a highly proactive personality, they usually take the initiative to explore and influence their environment, reflect on their future self-development (Kim & Park, 2017), and gradually form a sense of continuity and identity with themselves at different times. This means that for these college students, their future self is not a stranger, but rather a continuous whole, indicating a high degree of future self-continuity (F. Zhang et al., 2026). Furthermore, existing research has shown that the more an individual anticipates the future and the more positive their attitude, the stronger the connection between their present self and their future self (Hong et al., 2024; Pan et al., 2026). Individuals with a highly proactive personality are more willing to view the world with a positive outlook (D. L. Yang & Yang, 2026), have a clear understanding and pursuit of the future, and tend to closely connect their present with the future (Xue et al., 2023), actively influencing and changing the environment to achieve their predetermined goals (Grekin et al., 2025). Therefore, the second hypothesis of this study is proposed:

**Hypothesis** **2.** *Proactive personality of college students is positively related to their future self-continuity.*

From the perspective of social cognitive career theory (Lent et al., 2002; Lent & Brown, 2019), future self-continuity is not merely a psychological representation of time; it is actually the cognitive engine and motivational anchor for an individual’s career development (Chishima & Wilson, 2021). It fundamentally drives the construction and deepening of professional identity by reshaping an individual’s sense of self-efficacy, outcome expectations, and goal setting. College students with high future self-continuity use the clarity of their future self to combat present confusion and leverage the allure of future rewards to drive present engagement (Hong et al., 2024). They are not only “studying” a major, but also “rehearsing” a life. This future-oriented approach, characterized by high self-efficacy, positive outcome expectations, and continuous proactive exploration, forms the solid core of professional identity (Xue et al., 2023). Therefore, future self-continuity is not only the result of proactive personality driving career exploration but also a prerequisite for transforming external proactive behavior into committed professional identity (Shen et al., 2024). This means that a proactive personality can effectively foster a high level of professional identity when students clearly “see” and “connect” to their future professional selves (Rutt & Löckenhoff, 2016). Taken together, the third hypothesis of this study is proposed:

**Hypothesis** **3.** *Future self-continuity of college students mediates the relationship between proactive personality and professional identity.*

### 2.3. The Mediating Role of Career Calling

Career calling refers to the strong and meaningful passion that an individual feels for a particular professional field (Dobrow & Tosti-Khabras, 2011). At the beginning of this century, it was considered to be “the call of God” and was more likely to be interpreted as an external call for individuals to do something (Dalton, 2001). However, as research deepened, this calling was considered to be based on the country, society, and family. In the specific psychological field, career calling often dilutes its external appeal and gradually focuses on a certain inner calling (Schermer Sellers et al., 2005). Many prior studies have shown that individuals with a higher sense of career calling show concern for the value and contribution of their work to society, and experience the joy and self-realization brought by work (Neubert & Halbesleben, 2015).

A large number of previous studies have confirmed that proactive personality is the core driving force behind the formation of an individual’s career calling (Hanan et al., 2021; Yu et al., 2023). Individuals with a highly proactive personality are not content with passively adapting to their environment, but instead actively adjust task boundaries and cognitive frameworks through job crafting, constructing unique social meaning in ordinary work. This autonomous behavior pattern precisely satisfies basic psychological needs such as competence, autonomy, and belonging, transforming external professional requirements into intrinsic value pursuits (Gong & You, 2024). At the same time, their long-term future orientation prompts individuals to deeply link personal development with social well-being, thereby generating a sense of “calling” that transcends personal interests. This means that the stronger a person’s proactive personality, the more they can imbue their profession with transcendent value through strategic initiatives (Ma et al., 2024), thereby forming a firm and lasting sense of professional mission. Therefore, the fourth hypothesis of this study is proposed:

**Hypothesis** **4.** *Proactive personality of college students is positively related to their career calling.*

Social cognitive career theory emphasizes that career calling, as a profound intrinsic recognition of the social value of a profession (Bott & Duffy, 2015), is a key driving mechanism that promotes the transformation of professional identity from role-playing to identity recognition. Specifically, it endows professions with lofty goals through meaning construction, surpasses mere instrumental rationality (Hart & Hart, 2023), and thereby eliminating role ambiguity and establishing clear identity boundaries at the cognitive level. Meanwhile, career calling can inspire a sense of autonomy and competence in individuals, satisfy deep psychological needs, and foster strong emotional attachment and resilience, enabling professional identity to go beyond short-term utilitarian exchanges (Lobene & Meade, 2013). In addition, career calling is not merely an individual’s aspirations for their future profession; it is the core psychological bond connecting positive personality traits (behavioral tendencies) and professional identity (status) (Duffy & Dik, 2013). It elevates the individual’s proactive exploration into a life meaning that surpasses personal interests, thus completing the transformation of professional identity from “passive adaptation” to “active construction”. Therefore, the final hypothesis of this study is proposed:

**Hypothesis** **5.** *Career calling of college students mediates the relationship between proactive personality and professional identity.*

## 3. Methods

### 3.1. Participants and Procedure

This study follows the principle of convenience sampling and selects 500 full-time college students from a key municipal normal university in Beijing, China, as the research sample. With the support of the school’s student management department, the research team distributed questionnaire links to selected students through the WeChat platform. To ensure ethical compliance, this study prominently included a confidentiality statement and academic use commitment on the questionnaire’s opening page: participants were clearly informed that “all data will be used solely for academic research, and the researchers promise to strictly protect personal information and will not conduct any form of identity tracking or commercial disclosure,” and were required to confirm their consent before answering.

At the same time, the questionnaire was de-identified throughout, removing all sensitive fields that could potentially reveal identity (such as name, contact information, etc.). This process not only standardizes data collection channels, but also effectively protects the privacy rights of participants through institutional ethical commitments. In addition, to further reduce the potential impact of common method bias (Saunders et al., 2016; Su et al., 2022), this study conducted three questionnaire surveys on selected college students. In the first survey, college students were asked to report their proactive personality traits and demographic information. In the second survey, college students were asked to complete a questionnaire on future self-continuity and career calling. In the third survey, college students were invited to complete a questionnaire about their professional identity.

After excluding invalid questionnaires—specifically those that could not be matched, contained missing answers, or exhibited obvious patterns in responses (such as all questions receiving identical answers)—this study ultimately secured 382 valid samples, achieving a response rate of 76.4%. The final sample included 163 males (42.67%) and 219 females (57.33%). The college students participating in the survey were evenly distributed across different grades, with 85 first-year students (22.34%), 110 second-year students (28.77%), 112 third-year students (29.32%), and 75 fourth-year students (19.63%). In addition, 183 students (47.9%) had rural household registration, and 199 students (52.1%) had urban household registration. Regarding family structure, 167 students (43.7%) were only children, meaning they had no siblings, while 215 students (56.3%) were not only children, indicating they had at least one sibling.

### 3.2. Measures

The scales used in the current study were all selected from classic domestic and international literature and from mature scales that had been developed. Meanwhile, for scales developed from Western contexts, this study employed standard translation/back-translation procedures to ensure the scales’ cross-cultural adaptability (Brislin, 1983). The specific steps are as follows: First, two psychology graduate students who were proficient in both Chinese and English independently translated the English scale into Chinese. Then, a native English speaker who was familiar with psychological terminology translated the Chinese scale back into English. Finally, the research team compared the original English scale with the back-translated English scale, discussed and adjusted the differences to ensure that the Chinese scale was semantically consistent with the original scale. All items were scored using a 5-point Likert scale, ranging from “1 = completely disagree” to “5 = completely agree”.

#### 3.2.1. Proactive Personality Scale

The Proactive Personality Questionnaire, developed by Parker (1998), was used to measure the proactive personality of college students. This scale consists of 6 items and has been validated in the Chinese context (Ma et al., 2024; Y. Zhang et al., 2023). Example items include: “If I believe in an idea, no obstacle can stop me from making it happen”, “I like to challenge the status quo”, and “I am always looking for new ways to make my life better”. The Cronbach’s α for this scale in this study was 0.902.

#### 3.2.2. Future Self-Continuity Scale

The Future Self-Continuity Questionnaire, developed by Sokol and Serper (2020), was used to measure the future self-continuity of college students. This scale consists of 10 items and has been validated in the Chinese context (Wei et al., 2025). Example items include: “To what extent can you vividly imagine yourself ten years from now”, “To what extent can you like yourself ten years from now”, and “To what extent can you like the way you do things ten years from now”. The Cronbach’s α for this scale in this study was 0.897.

#### 3.2.3. Career Calling Scale

The Career Calling Scale, which was developed and verified by C. Zhang et al. (2015) in the Chinese context, was used to test the career calling of college students. This scale contains 11 items, with example items including: “I want to find meaning in life through my career”, “I feel an invisible force driving me to pursue a certain career”, and “I want to do something beneficial for society through my career”. The Cronbach’s α for this scale in this study was 0.937.

#### 3.2.4. Professional Identity Scale

The Professional Identity Scale, which was developed and verified by X. Liu et al. (2023) in the Chinese context, was introduced to test the professional identity of college students. This scale consists of 10 items on “high career satisfaction” and 3 items on “strong career interest, high career strength, and good career prospects”. The Cronbach’s α for this scale in this study was 0.918.

### 3.3. Statistical Analysis Strategies

This study mainly used SPSS 26.0 and Amos 26.0 statistical analysis software for data analysis and hypothesis verification. The specific testing steps are as follows: First, the reliability and validity of the sample data were tested using coefficient α, internal consistency coefficients, KMO value, and confirmatory factor analyses (CFAs), following the procedures outlined by Green and Yang (2015) and Shrestha (2021). Next, the common method bias of the sample data was tested using Harman’s single-factor test, as recommended by Zhou and Long (2004). Subsequently, descriptive statistics and correlation analysis were used to conduct a preliminary analysis of the relationships between proactive personality, future self-continuity, career calling, and professional identity, in line with the practices reported by T. A. Brown and Moore (2012). Finally, we employed multiple linear regression and the PROCESS 4.2 macro to examine the influence of proactive personality on professional identity, particularly examining the mediating roles of future self-continuity and career calling in this influencing process, following the methodologies described by Hayes (2013).

## 4. Results

### 4.1. Reliability and Validity Testing

In order to test the reliability of the main variables designed in this study, including proactive personality, future self-continuity, career calling, and professional identity, SPSS 26.0 analysis software was used for analysis. The test results show that the coefficient α and internal consistency coefficients of the scales of each variable used in this study are all greater than 0.800, and the scale reliability is good (Green & Yang, 2015). To further assess the suitability of our data for factor analysis, this study conducted Bartlett’s sphericity test, which examines the null hypothesis that the correlation matrix is an identity matrix, indicating no correlation between variables. The significant result of Bartlett’s test (*p* < 0.001) supported the rejection of this null hypothesis, confirming the presence of inter-correlations among variables and thus the appropriateness of factor analysis (Bartlett, 1954). On this basis, the Kaiser–Meyer–Olkin (KMO) measure of sampling adequacy for the questionnaires is 0.906, 0.874, 0.898 and 0.917, respectively, all above 0.800 (Shrestha, 2021), demonstrating good construct validity.

To test the discriminant validity of proactive personality (6 indicators), future self-continuity (10 indicators), career calling (11 indicators), and professional identity (13 indicators), this study first conducted a series of confirmatory factor analyses (T. A. Brown & Moore, 2012), the results of which are shown in Table 1. The analysis results are as follows: x^2^/*df* = 2.47, RMSEA = 0.062, CFI = 0.914, TLI = 0.902, SRMR = 0.061. It can be seen that all the fit indices of the scales used in this study meet the evaluation criteria. The model constructed in this study, which includes proactive personality, future self-continuity, career calling, and professional identity, has a good fit effect, and the discriminant validity among the variables is also satisfactory.

### 4.2. Common Method Bias Testing

Although this study employed a three-stage data collection approach, the fact that all data were obtained through self-assessment by participating students may have led to common methodological bias in the results. Hence, before formal data analysis, this study employed the Harman’s single-factor method to assess potential common method bias. The results show that the first factor explained 38.89% of the variance, which is below the 40% threshold (Zhou & Long, 2004). Thus, the common method bias problem in this study is not significant.

### 4.3. Descriptive Statistics and Correlations Testing

This study conducted correlation analyses to examine the relationships among proactive personality, future self-continuity, career calling, and professional identity. The results are shown in Table 2. It can be found that the correlation coefficients among the main variables involved in this study range from 0.499 to 0.620, and all are significant at the 0.01 level. Hence, there is a significant positive correlation between each pair of variables, which also provides preliminary evidence for the five hypotheses proposed.

### 4.4. Hypotheses Testing

This study employed regression analysis using SPSS 26.0 software to examine the relationships among the main variables. The results, presented in Table 3, indicate that in Model 6—after controlling for demographic variables such as sex (male/female) and grade level—proactive personality significantly and positively predicts professional identity (β = 0.659, *p* < 0.001). This suggests that for every standard deviation increase in proactive personality, there is a corresponding 0.659 standard deviation increase in professional identity, holding other variables constant. This effect size is considered large in the context of social science research (Cohen, 1988; Gignac & Szodorai, 2016), supporting Hypothesis 1 that proactive personality positively relates to professional identity.

As shown in Model 2, after controlling for variables such as college students’ sex and grade, proactive personality has a significant positive predictive effect on future self-continuity (β = 0.474, *p* < 0.001). Specifically, for every standard deviation increase in proactive personality, future self-continuity increases by approximately 0.474 standard deviations, and the effect is significant (*p* < 0.001), indicating that proactive personality is an important factor influencing future self-continuity, supporting Hypothesis 2. As shown in Model 4, after controlling for variables such as sex and grade level among college students, proactive personality has a significant positive predictive effect on career calling (β = 0.633, *p* < 0.001). Similarly, for every standard deviation increase in proactive personality, career calling rises by approximately 0.633 standard deviations, with this effect being highly significant (*p* < 0.001), thus indicating that proactive personality is a key factor in career calling and supporting Hypothesis 4.

As can be seen from Model 7, when proactive personality and future self-continuity are included in the regression equation, future self-continuity has a significant positive predictive effect on professional identity (β = 0.307, *p* < 0.001), while the positive predictive effect of proactive personality on professional identity is significantly smaller and still significant (β = 0.514, *p* < 0.001). Therefore, future self-continuity mediates the predictive effect of proactive personality on professional identity. Meanwhile, as shown in Model 8, when proactive personality and career calling were included in the regression equation, career calling had a significant positive predictive effect on professional identity (β = 0.301, *p* < 0.001), while the positive predictive effect of proactive personality on professional identity became significantly smaller (β = 0.469, *p* < 0.001). Hence, career calling mediates the predictive effect of proactive personality on professional identity.

Furthermore, to ensure the consistency and stability of the analysis results, this study employed the Bootstrap method (Hayes, 2013), drawing 5000 Bootstrap samples to verify the parallel mediating role of future self-continuity and career calling between proactive personality and professional identity. The analysis results are shown in Table 4. As indicated, the indirect effect of proactive personality influencing professional identity through future self-continuity has a confidence interval of [0.131, 0.275] at the 95% level, excluding 0, and the direct effect has a confidence interval of [0.287, 0.506] at the 95% level, also excluding 0. At the same time, the indirect effect of proactive personality in influencing professional identity through a sense of career mission has a 95% confidence interval of [0.093, 0.336], excluding 0, and the direct effect has a 95% confidence interval of [0.244, 0.503], also excluding 0. Taken together, both Hypothesis 3 and Hypothesis 5 are fully supported.

## 5. Discussion

Based on social cognitive career theory, this study found that proactive personality has a significant positive impact on professional identity, both directly and indirectly through career calling and future self-continuity. Specifically, given that the indirect effect via career calling (β = 0.213) was slightly larger than via future self-continuity (β = 0.196), this suggests that career calling may play a more central role in mediating the relationship between proactive personality and professional identity in the Chinese cultural context. This finding aligns with previous research indicating that career calling is a powerful motivator for individuals to pursue meaningful careers (Duffy & Dik, 2013), and extends social cognitive career theory by highlighting the importance of internal motivation (i.e., career calling) in shaping professional identity, especially among Chinese college students who are deeply influenced by Confucian values such as the pursuit of meaning and purpose in life.

### 5.1. Theoretical Implications

Firstly, this study expands the research on the antecedents of professional identity, revealing the cross-situational influence mechanism of proactive personality. Specifically, based on social cognitive career theory (Lent & Brown, 2019; Lent et al., 2002), this study constructs a dual-mediation model of “proactive personality → future self-continuity/career calling → professional identity” (see Figure 1 for a detailed framework), revealing the intrinsic path by which personality traits influence professional identity through cognitive construction. This also breaks through the limitations of traditional professional identity research that only focuses on demographic variables or occupational characteristics (S. D. Brown & Lent, 2017), and for the first time verifies the positive predictive role of proactive personality on professional identity in the field of higher education. This finding not only enriches the research on antecedent variables of professional identity (Seibert et al., 2001; Barbour & Lammers, 2015), but also provides a theoretical basis for understanding the cross-situational effects of proactive personality (Wang et al., 2025; Yu et al., 2023), such as the transfer from professional behavior to the field of higher education.

Secondly, this study incorporates future self-continuity and career calling into the same theoretical framework, revealing their differentiated mediating roles in the relationship between proactive personality and professional identity. On the one hand, future self-continuity, as an individual’s cognition of the consistency between the “present self” and the “future self” (Han et al., 2025; F. Zhang et al., 2026), has been demonstrated to be an important cognitive mechanism by which proactive personality influences professional identity. On the other hand, career calling, as an altruistic career motivation (Hanan et al., 2021; Yu et al., 2023), has been verified as an emotional bridge connecting personality traits and professional identity. This dual-mediation model not only responds to the academic call for “how proactive personality influences professional identity through both cognitive and emotional channels,” but also further enriches the relevant research on future self-continuity and career calling (Chishima & Wilson, 2021; Rutt & Löckenhoff, 2016).

Thirdly, the findings of this study significantly advance social cognitive career theory by providing empirical evidence for the mediating roles of career calling and future self-continuity in the relationship between proactive personality and professional identity. While social cognitive career theory has primarily focused on self-efficacy, outcome expectations, and goals in career choices and development (Lent et al., 2002; Lent & Brown, 2019), our findings highlight the crucial importance of internal motivation and future orientation, especially among proactive individuals, in shaping professional identity. Furthermore, by examining this relationship within the Chinese cultural context, our study challenges the conventional view that professional identity is solely influenced by external factors, and underscores the significant impact of individual differences. This collectively suggests that future social cognitive career theory research should pay more attention to the role of individual differences in internal motivation and future orientation (S. D. Brown & Lent, 2017), explore their interaction with environmental factors, and investigate cultural moderators to better understand how proactive personality shapes professional identity across diverse cultural settings (Jiang & Zhang, 2012).

### 5.2. Practical Implications

This study offers significant practical insights into how to effectively enhance college students’ professional identity, particularly how to substantially improve their professional identity within educational settings. To begin with, from the perspective of proactive personality, in order to enhance college students’ professional identity, instructors should praise and affirm the various characteristics of proactive personality, and continuously inspire students to take positive actions to influence their surrounding environment (Ma et al., 2024; Wang et al., 2025). Meanwhile, teachers should focus on improving students’ classroom participation and actively encourage them to participate in various academic competitions, research projects, and other practical activities (De Clercq & Pereira, 2023). By participating in these activities, students can deepen their experience and understanding of proactive behavior in practice, further strengthen their sense of initiative, and subtly shape and consolidate their proactive personality tendencies, ultimately enhancing their professional identity.

Furthermore, the mediation effect analysis of future self-continuity shows that when students believe that their future self is highly consistent with their present self, their level of professional identity is often also high. Individual career development is a gradual and interconnected process (Chishima & Wilson, 2021; Kim & Park, 2017). Learning and growth during the student stage are closely linked and reinforce future career development. The future self is formed based on the continuous development and evolution of the present self. Therefore, teachers should actively play a guiding role and encourage students to envision the future from multiple perspectives, including personal growth planning, career development vision, and alignment with national development strategies, thereby enhancing students’ positive expectations for the future (X. Liu et al., 2023). For example, special activities such as “Write a letter to your future self” and “Draw a portrait of your future self” can be organized to effectively improve students’ continuity of their future self-continuity through these specific and creative methods.

Finally, the mediating role of career calling suggests that a variety of teaching methods and forms should be employed to consistently stimulate students’ career calling. On the one hand, it is essential to persistently strengthen educational guidance, integrating professional mission content into ideological and political courses, and incorporating professional values and contributions to the industry into professional teaching (Dalton, 2001). On the other hand, practical experience should be emphasized, encouraging students to participate in practical activities and projects to understand the value of their profession, while also establishing a professional practice mentoring system to provide one-on-one guidance for college students (Neubert & Halbesleben, 2015). In addition, universities should create a positive atmosphere, subtly instill students’ career ideals through campus cultural activities and role models, and establish honorary awards to recognize students with a strong career calling (Duffy & Dik, 2013). These awards not only affirm and encourage students’ past efforts, but also motivate and guide their future career development, stimulating their intrinsic motivation and encouraging them to work harder to pursue their career goals.

### 5.3. Limitations and Future Research

Having discussed the theoretical and practical implications of our findings, it is now crucial to acknowledge the limitations of our study, which provide important avenues for future research. For instance, the data collected in this study were obtained through self-assessment by the participating college students, which may be subject to common method bias, affecting the accuracy of the research results. Therefore, future scholars could consider using a paired approach of teacher evaluation and student self-evaluation to measure relevant variables, in order to minimize such problems and further improve the accuracy of research conclusions. Meanwhile, in terms of sampling methods, this study used convenience sampling, and the sample came from only one normal university in Beijing. This may limit the general applicability of the research results, and the results may not be directly generalized to all Chinese college students. Therefore, future research should consider employing a broader sampling strategy, including samples from multiple institutions and regions, to further validate and expand the findings of this study. In addition, this study mainly constructed and verified a theoretical model based on a sample of Chinese college students, and its conclusions may be influenced by specific cultural backgrounds, including collectivist orientation and educational values (Long et al., 2026; J. Zhang & Han, 2023). The relationship between proactive personality and professional identity may differ across different cultural backgrounds. Future research needs to conduct cross-cultural comparisons to verify the universality of theoretical models or reveal cultural specificities in order to enhance the applicability of research conclusions. Finally, this study only explored the influencing factors of professional identity from the perspectives of individual traits and cognition, and did not involve other factors that may affect professional identity, such as student behavior (Barbarà-i-Molinero et al., 2017; Barbour & Lammers, 2015; Ma et al., 2024). Future research could be expanded to explore the impact of student behavior, environmental factors, and other aspects on professional identity in order to reveal the influencing factors of professional identity more comprehensively.

## 6. Conclusions

In summary, this study focuses on Chinese college students as the research participants and explores the relationship between their proactive personality and professional identity, as well as the interaction and influence mechanism between future self-continuity and career calling in this context. The findings verified that the proactive personality of college students has a significant positive impact on their professional identity; future self-continuity and career calling both play a partial mediating role in the relationship between proactive personality and professional identity. These findings not only deepen the scholarly understanding of proactive personality by elucidating its role in shaping college students’ professional identity, but also offer practical insights for educational interventions aimed at fostering and strengthening professional identity among college students.

## Figures and Tables

**Table 1:** Results of confirmatory factor analyses.

Model	Factor	χ^2^/*df*	CFI	TLI	RMSEA	SRMR
Four-factor model	PP, FSC, CC, PI	2.47	0.914	0.902	0.062	0.061
Three-factor model	PP, FSC + CC, PI	3.37	0.851	0.844	0.079	0.104
Two-factor model	PP + FSC + CC, PI	3.62	0.837	0.785	0.083	0.127
Single-factor model	PPF + FSC + CC + PI	4.07	0.801	0.798	0.090	0.109

Note: N = 382; PP = proactive personality; FSC = future self-continuity; CC = career calling; PI = professional identity; Ideal model-fit indicators are: χ^2^/*df* < 3, CFI > 0.9, TLI > 0.9, RMSEA < 0.08, SRMR < 0.08.

**Table 2:** Descriptive statistics and correlation coefficients of variables.

Variables	Means	SD	1	2	3	4
1. Proactive Personality	3.79	0.438				
2. Future Self-continuity	3.39	0.364	0.499 **			
3. Career Calling	3.71	0.422	0.619 **	0.563 **		
4. Professional Identity	3.50	0.431	0.620 **	0.538 **	0.572 **	1

Note: N = 382; ** *p* < 0.01.

**Table 3:** Regression analysis results of main variables.

Variables	FSC		Career Calling		Professional Identity			
	Model 1	Model 2	Model 3	Model 4	Model 5	Model 6	Model 7	Model 8
Sex	0.248 ***	0.019	0.264 ***	−0.041	0234 ***	−0.084 *	−0.090 *	−0.072
Grade	0.156 **	0.045	0.173 **	0.025	0.170 **	0.016	0.002	0.009
PP		0.474 ***				0.659 ***	0.514 ***	0.469 ***
FSC				0.633 ***			0.307 ***	
Career Calling								0.301 ***
R^2^	0.102	0.251	0.120	0.385	0.101	0.389	0.460	0.445
ΔR^2^		0.149		0.265		0.288	0.071	0.056
F	21.64 ***	42.27 ***	25.81 ***	78.87 ***	21.38 **	80.34 ***	80.21 ***	75.59 ***

Note: N = 382; PP = proactive personality; FSC = future self-continuity; * *p* < 0.05, ** *p* < 0.01, *** *p* < 0.001.

**Table 4:** Results of mediation of future self-continuity and career calling.

Mediator Variable	Type of Effect	Effect	SE	95% CI	
				Lower	Upper
Future Self-continuity	Indirect effect	0.196	0.044	0.131	0.275
	Direct effect	0.391	0.063	0.287	0.506
	Total effect	0.582	0.051	0.482	0.681
Career Calling	Indirect effect	0.213	0.067	0.093	0.336
	Direct effect	0.374	0.069	0.244	0.503
	Total effect	0.587	0.051	0.483	0.688

Note: N = 382; SE = Standard Error; CI = confidence interval.

## Data Availability

Data supporting reported results are available from the authors on request.
